# Validation of Endogenous Control Genes for Gene Expression Studies on Human Ocular Surface Epithelium

**DOI:** 10.1371/journal.pone.0022301

**Published:** 2011-08-03

**Authors:** Bina Kulkarni, Imran Mohammed, Andrew Hopkinson, Harminder Singh Dua

**Affiliations:** 1 Division of Ophthalmology and Visual Sciences, Queen's Medical Centre, Nottingham, United Kingdom; 2 Institute for Translational Medicine and Therapeutics, Department of Pharmacology, University of Pennsylvania School of Medicine, Philadelphia, Pennsylvania, United States of America; University of Memphis, United States of America

## Abstract

**Purpose:**

To evaluate a panel of ten known endogenous control genes (ECG) with quantitative reverse transcription PCR (qPCR), for identification of stably expressed endogenous control genes in the ocular surface (OS) epithelial regions including cornea, limbus, limbal epithelial crypt and conjunctiva to normalise the quantitative reverse transcription PCR data of genes of interest expressed in above-mentioned regions.

**Method:**

The lasermicrodissected (LMD) OS epithelial regions of cryosectioned corneoscleral buttons from the cadaver eyes were processed for RNA extraction and cDNA synthesis to detect genes of interest with qPCR. Gene expression of 10 known ECG—glyceraldehyde-3-phosphate dehydrogenase (GAPDH), beta actin (ACTB), peptidylprolyl isomerase (PPIA), TATA-box binding protein (TBP1), hypoxanthine guanine phosphoribosyl transferase (HPRT1), beta glucuronidase (GUSB), Eucaryotic 18S ribosomal RNA (18S), phosphoglycerate kinase (PGK1), beta-2-microglobulin (B2M), ribosomal protein, large, P0 (RPLP0)—was measured in the OS epithelial regions by qPCR method and the data collected was further analysed using geNorm software.

**Results:**

The expression stability of ECGs in the OS epithelial regions in increasing order as determined with geNorm software is as follows: ACTB<18S<TBP<B2M<PGK1<HPRT1<GUSB<GAPDH<PPIA-RPLP0. In this study, geNorm analysis has shown the following ECGs pairs to be most stably expressed in individual OS epithelial regions: HPRT1-TBP in cornea, GUSB-PPIA in limbus, B2M-PPIA and RPLP0-TBP in LEC and conjunctiva respectively. However, across the entire ocular surface including all the regions mentioned above, PPIA-RPLP0 pair was shown to be most stable.

**Conclusion:**

This study has identified stably expressed ECGs on the OS epithelial regions for effective qPCR results in genes of interest. The results from this study are broadly applicable to quantitative reverse transcription PCR studies on human OS epithelium and provide evidence for the use of PPIA-RPLP0 ECGs pair in quantitative reverse transcription PCR across the OS epithelium.

## Introduction

In recent years' molecular studies on ocular surface (OS) epithelium are gaining popularity especially with respect to limbal stem cells (LSC), antimicrobial peptides, allergic and inflammatory eye diseases, microbial infections, keratoconjunctivitis sicca or dry eyes, pterygium, and ocular surface wound healing [1]–[4]. The commonly performed method in these studies is quantitative reverse transcription PCR (qPCR) for gene expression analysis. The high sensitivity and specificity of qPCR has made it a gold standard validation tool for gene expression studies such as microarrays, which have high throughput but low sensitivity [5], [6]. Commonly, qPCR experiments involve comparison between the samples, therefore uniformity of the samples is crucial [7]. However, several determinants such as RNA quantity, quality, degradation of the samples, presence of contaminants in the reagents or the samples and differences in the reverse transcription efficiencies may generate inaccurate qPCR data if the gene expression was normalised to input RNA. To overcome inaccuracies due to the variability in samples, normalisation of genes of interest in the samples has been performed with a reference gene, where the reference gene RNA is reverse transcribed with the RNA of the gene of interest. However, studies have shown variations in expression of known constitutively expressed reference genes in different experimental conditions and samples [8], [9]. So far the expression of ideal reference gene(s) that remains stably expressed in different experimental conditions and in different tissues has not been proven [10], [11]. Vandesompele et al (2002) have proposed a normalisation strategy which involves use of multiple constitutively expressed endogenous genes in the samples. Geometric mean of these genes is calculated with geNorm algorithm (Primer Design, Ltd., Southampton University, UK) and the gene of interest is normalised with the normalisation factor (NF) calculated with this geometric mean [12]. Several studies performed in different experimental conditions [13], [14] and with different samples [15], [16] have demonstrated effectiveness of this normalisation strategy. For tissue samples obtained by laser microdissection, the identification of stable endogenous control genes (ECGs) is particularly crucial for reliable normalisation, due to the fact that minimal amount of RNA yield from these samples makes optimisation of samples by quantification of RNA difficult [9]. Pubmed search for qPCR studies on OS epithelium revealed 46 qPCR studies from 2002 to 2010. Genes of interest in 28 of these studies was normalised using GAPDH, 7 using ACTB, 5 with 18S, 2 studies with cyclophillin, and one study each with HPRT1 and B2M. GAPDH and ACTB were predominantly preferred ECGs in the OS epithelial regions. However, several studies have demonstrated unstable expression of these genes (GAPDH, ACTB) in various tissues and experimental conditions [9], [17]–[19]. Despite the widespread application of qPCR technology in OS, studies to determine the expression of stable ECGs in the human OS epithelium have not yet been performed. With the advent of laser microdissection (LMD) technique for sample collection in ophthalmology [2], [20], [21] identification of stably expressed ECGs has become crucial for accurate normalisation strategy due to difficulties in quantifying and maintaining uniformity of RNA concentrations in these samples [22]. The aim of this study was to quantify the gene expression of ten known ECG with Taqman gene expression assays across the LMD samples of OS epithelial regions of cornea, limbus, limbal epithelial crypt (LEC) and conjunctiva. The genes belonged to the following functional categories: i) genes involved in metabolism: GAPDH, HPRT1, RPLP0, PGK1, GUSB; ii) genes involved in transcription: TBP; iii) genes involved in cell structure and motility: ACTB; iv) genes involved in protein folding: PPIA; v) gene involved in immune response: B2M and vi) gene involved in synthesis of ribosomal protein: 18S. The qPCR data in this study was analysed with GeNorm algorithm to determine the expression stability (M) and calculate the normalisation factor (NF) of the most stable pair of ECGs. This would enable efficient normalisation of genes of interest across the OS epithelial regions.

## Methods

### Tissue samples

This study was conducted with the approval of Nottingham Research Ethics Committee (REC No: OY030202). The protocol was consistent with Tenets of Declaration of Helsinki. Informed written consent was obtained from relatives of all the deceased donors regarding eye donation for research. Three pairs of cadaver eyes were harvested within 48 hours of death under aseptic conditions using conventional techniques to maintain RNA viability. The inclusion criteria were donor age between 20 to 70 years, donors of either sex; eyes with undamaged OS epithelium confirmed by physical examination with a dissecting microscope and no history of past ocular disease or intervention, as ascertained from the case notes.

The samples used in this study were obtained by laser microdissection of frozen tissue sections of corneoscleral button with the overlying corneal, limbal and conjunctival epithelium dissected from freshly obtained cadaver eyes [2]. The corneoscleral button was placed in a petri dish with PBS to prevent drying of the OS. The corneoscleral button was cut radially into eight triangular segments. Handmade aluminium foil cups of approximately 20 mm diameter were filled with an embedding medium optimum temperature compound (OCT, Emitech Ltd, East Sussex, England). Each triangular segment of corneoscleral button was then oriented in the OCT compound, in such a way that one of the long edges of the triangle was parallel to the surface of the OCT medium and short edge perpendicular to this edge. The aluminium foil caps containing the tissue segments were immersed in isopentane bath pre-chilled (frozen) in liquid nitrogen for gradual freezing of the tissue segments to prevent freeze fractures in the tissue sections. These frozen tissue blocks were stored at −80°C for future cryosectioning.

The corneoscleral cryosections of approximately 6 µm thickness were examined under a light microscope to assess the quality of the epithelium. These OS epithelial cryosections consisted of 2–3 cell thick layer of compactly arranged epithelial cells, due to which the individual counting of the epithelial cells was not possible. However, the area in µm^2^of lasermicrodissected epithelial tissue obtained was measured. It was approximately 100,000 µm^2^ for each laser microdissected OS epithelial samples catapulted in the collection tube to maintain the uniformity of the samples collected.

Real time PCR of each biological replicate for OS epithelial region was performed in triplicates to determine the reproducibility of the samples and the reliability of the experiments. The extracted RNA was quantified with Agilent Pico assay 6000 chip.

The subjectivity in the sampling during laser microdissection was controlled by identifying the epithelial regions of OS such as cornea, limbus, LEC, and conjunctiva by consistent easy to identify anatomical landmarks. The basal layer of the cornea is straight due to the support of the underlying Bowman's membrane which is an acellular refractile layer. This layer is easily demarcated in wet stained or unstained histological preparations.

The corneal epithelium termination and the beginning of the limbal epithelium are marked with termination of bowman's membrane which is easy to identify on the histological sections. The limbal epithelium has wavy appearance and is approximately 10–12 cell thickness unlike corneal epithelium which has uniform thickness.

Microscopic examination of limbal stroma reveals presence of blood vessels in this region compared to the corneal stroma which is an avascular structure.

The conjunctival epithelium can be identified separately to limbus with the following histological features of these OS epithelial regions: The junction between limbus and conjunctiva is marked as a constriction in the epithelium, due to change in the limbal epithelial thickness from 10–12 cells to 2–5 cells thick stratified columnar conjunctival epithelium. The epithelial surface of the conjunctiva is irregular and ragged. Presence of goblet cells in the epithelium is also a feature for identification and confirmation of the conjunctival tissue. Compared to limbal stroma the conjunctival stroma has dense infiltration of cells lymphocytes along with the presence of blood vessels, nerves and accessory lacrimal glands with distinct amorphous material in the central lumen. In a previous study, Prof H S Dua has identified a unique structure at the limbus, termed as the Limbal Epithelial Crypt [23]. LEC is a solid cord of cells that extends from the peripheral aspect of the undersurface of limbal palisade of Vogt into conjunctival stroma, either parallel or perpendicular to these palisades. LECs vary in size, shape and in their location around the limbus. The junctional zones in the OS epithelium were avoided during LMD.

### RNA Extraction

Total RNA extraction of LMD tissue was performed with RNeasy Micro kit, according to manufacturer's protocols (QIAGEN, West Sussex, UK). Each sample was made up to 350 µl with RLT buffer. The samples were briefly vortexed followed by addition of 350 µl of 70% v/v ethanol to promote binding of RNA to the RNeasy MinElute membrane. The reaction consisting of sample, RLT buffer and ethanol was mixed by repeated pipetting, before transferring to a spin column and centrifuged briefly at ≥10,000 rpm, following which the flow through was discarded.

350 µl of RW1 buffer was added to the spin column and centrifuged as above. DNase treatment of RNeasy spin columns was carried out for 15 minutes with 10 µl DNase1 added to 70 µl RDD buffer. The wash step with RW1 buffer was repeated and the collection tube changed.

500 µl of RPE buffer was pipetted onto the spin column and centrifuged at 10,000 rpm, for 15 seconds. 80%, of 500 µl ethanol was added to the spin column and centrifuged for 2 minutes at 10,000 rpm. The flow through and the tube were discarded. The opened spin column was thoroughly dried by centrifuging at the full speed for 5 minutes.

Finally, 14 µl of RNase free water was pipetted into the centre of the silica-gel membrane and centrifuged at maximum speed for 1 minute to elute 12 µl RNA.

The RNA tubes were stored at −80°C until further use. RNA quantity and quality were measured with nano6000 Picochip on a Picoassay 2100 Bioanalyzer (Agilent Technologies, USA).

### cDNA synthesis and amplification for real time PCR

The RNA samples from OS epithelial regions of three pairs of eyes were concentrated to 6 ng/5 µl each for cDNA synthesis. These RNA samples were processed with NuGEN WT-Ovation™ Pico RNA Amplification System (NuGen Technologies, Inc., San Carlos, CA) for first and second strand cDNA synthesis followed by amplification. The same set of cDNA samples were used to perform qPCR experiments for genes of interest and ECGs.

### First Strand cDNA synthesis

2 µl of First Strand Primer mix (NuGen Technologies, Inc., San Carlos, CA) A1 *ver* 3 was added to the 5 µl of RNA sample, and centrifuged. The tubes were placed in thermal cycler at 65°C for 2 minutes and then on ice. 3 µl of First Strand Master Mix (2.5 µl Buffer Mix A2 *ver* 3+0.5 µl Enzyme mix A3 *ver* 1) was then added to the above mentioned sample tubes and centrifuged. The tubes were again placed in thermal cycler at 4°C for 1 minute, 25°C for 10 minutes, 42°C for 10 min, 70°C for 15 min and 4°C thereafter to generate first strand cDNA.

### Second Strand cDNA synthesis

For the synthesis of second strand cDNA, 10 µl of Second Strand Master Mix (9.75 µl Buffer Mix B1 *ver* 3+0.25 µl Enzyme mix B2 *ver* 2) was added to the above first strand reaction tubes. The tubes were then centrifuged and placed in thermal cycler at 4°C for one min, 25°C for 10 min, 50°C for 30 min, 70°C for 5 min, 4°C thereafter.

### Purification of double stranded cDNA

To each of the second strand reaction tubes 1 µl of 50 ng/µl of yeast tRNA carrier was added. To this mix, 32 µl of the RNAClean® beads (NuGen Technologies, Inc., San Carlos, CA) was added and incubated at room temperature for 10 minutes. The tubes were placed on the magnet for 5 minutes to separate the beads. 45 µl of the binding buffer was removed to minimise the bead loss. The beads were washed thrice with 200 µl of freshly prepared 70% ethanol and allowed to dry for 15–20 minutes before SPIA™ amplification.

### SPIA™ amplification

This method was performed for rapid amplification of cDNA from LMD RNA samples, the amplification with this method occurred randomly throughout the whole transcriptome in the sample and not just at the 3′ end. To each second strand reaction tube containing dried beads 160 µl of SPIA™ master mix was added on ice and centrifuged. Each 160 µl reaction was halved and then placed in a thermal cycler at 4°C for 1 minute, 47°C for 60 min, 95°C for 5 min and 4°C thereafter. Following this the reaction tubes were spun and purified. The amplified cDNA samples (130–200 ng/µl) were optimised to a concentration of 30 ng/µl for qPCR of ECGs and genes of interest in the OS.

The contamination of the cDNA samples with genomic DNA was avoided by including additional steps in RNA and cDNA synthesis. According to recommendations by WT-Ovation™ Pico RNA Amplification System vs 1.0, Catalogue # 3300-12, user guide, DNase-treated RNA was used for synthesis of amplified cDNA to prevent contamination with the genomic DNA. Following SPIA amplification the amplified cDNA was purified with Zymo Research DNA Clean & ConcentratorTM-25. The purified cDNA was then assessed with Agilent assay to quantify and assess the quality of the cDNA and rule out contamination with genomic DNA.

### Real time PCR for quantification of reference genes

The relative quantification of ECGs in OS epithelial regions was performed with qPCR on Roche Lightcycler 480. Inventoried Taqman assays (Applied Biosystems, Foster City, CA) were used for selected genes of interest and endogenous controls genes (Table 1). Each reaction was performed in triplicate with final reaction volume of 15 µl. The reaction components for each well of the 384 well plate comprised of: 7.5 µl of TaqMan® Fast Universal PCR Master Mix (2×), No AmpErase® (Applied Biosystems, Foster City, CA Applied Biosystems), 0.75 µl of 20× Taqman Assay probes (Applied Biosystems), 5 µl cDNA 30 ng/µl concentration, 1.75 µl nuclease free water (Promega UK, Southampton, UK). The negative controls such as non template control and negative reverse transcriptase control were run on the PCR plate to rule out DNA cross contamination of the reagents and genomic DNA in the samples respectively. The cDNA prepared from Universal Human Reference RNA (Stratagene, La Jolla, CA) served as a positive control. Amplification was performed on the Light Cycler® 480 qPCR systems (Roche Diagnostics Ltd, UK) with the following thermal cycling parameters: One cycle of denaturation 95°C, hold: 10 minutes followed by 95°C cycle hold for 10 sec at ramp rate 4.4°C/s. The parameters for the third cycle were 60°C for 50 sec at 2.2°C/s. Fourth cycle of 72°C at 01 sec for 4.4°C/s was followed by the cooling step of 40°C at the ramp rate of 1.5°C/s for 10 sec.

**Table 1:** Endogenous control genes analysed with geNorm software in this study.

Gene symbol	Name	Accession number	Assays	Gene Functions
GAPDH	GAPD glyceraldehyde-3-phosphate dehydrogenase	NM_002046	Hs99999905_m1	Oxidoreductase in glycolysis and gluconeogenesis
B2M	Beta-2-microglobulin	NM_004048	Hs99999907_m1	MHC class l molecule
ACTB	Actin, beta	NM_001101	Hs99999903_m1	Cytoskeletal structural protein
PGK1	Phosphoglycerate kinase	NM_000291	Hs99999906_m1	Glycolytic enzyme
RPLP0	Ribosomal protein, large, P0	NM_001002	Hs99999902_m1	Structural component of 60S ribosomal unit
HPRT1	Hypoxanthine phosphoribosyl-transferase	NM_000194	Hs99999909_m1	Purine synthesis in salvage pathway
TBP	TATA box binding protein	M34960	Hs99999910_m1	General RNA polymerase II transcription factor
GUSB1	glucuronidase, beta	NM_000181	Hs99999908_m1	Hydrolytic enzyme involve in degradation of glycosaminoglycans
18S	Eucaryotic 18S ribosomal RNA	X03205	Hs99999901_m1	Component of ribosomal protein
PPIA	peptidylpropyl isomerase A	NM_021330	Hs99999904_m1	Involved in protein folding

### Data analysis

The gene expression levels or the crossing point (Cp) for each reaction of 10 ECGs was calculated (cp values) with relative quantification method [24] using Roche LC 480 software (Roche Diagnostics Ltd, West Sussex, UK) (Table 2). The average of triplicate values for each sample was then performed. These averaged Cp values were converted to linear values with delta-Ct formula Q = E^ΔCt^; where E is the efficiency of the gene, E = 2 approximately (precalculated efficiency value for inventoried TaqMan® Gene expression assays is 1.995) and ΔCt is the difference in the sample with the highest expression (minimum Cp value) in the data set with the Cp value of the sample studied [25]. The highest relative quantity of each gene was set to 1 with the above mentioned formula. The linear values for the ECG were inputted into GeNorm *ver.* 3.5 software (data not shown). The GeNorm VBA applet, which is compatible with Microsoft excel 2003 was downloaded from website: http://medgen.ugent.be/genorm/. GeNorm measures the expression stability of the panel of endogenous genes studied in the cDNA samples. Firstly, an array consisting of log_2_ transformed expression ratios of every combination of two internal control genes in each sample was created. Then standard deviation of all the values in the array for each combination of ECG was calculated. The expression stability or the M value for an endogenous gene was determined by calculating the arithmetic mean of the standard deviations for each ECG across the samples. The genes were then ranked according to their M values and the least stable ECG with the highest M value was eliminated from further analysis. A new set of M values was then generated for the remaining genes and the process of elimination of the gene with highest M value was repeated. This step wise elimination of the least stable reference gene was continued till two best reference genes were left at the end with similar M values, which ultimately could not be ranked any further. The normalisation factor (NF) was determined by calculating the geometric mean of the remaining stable ECGs (M value<1.5) divided by geometric mean of all NFs. To determine the number of stable ECGs needed for normalisation, a pairwise variation V_n/n+1_ was calculated for two sequential normalisation factors NF_n_ and NF_n+1_ for all the samples in an OS epithelial region. A graph of these V values was plotted to study the change in expression stability of the normalisation factors in comparison to the number of control genes. Variation larger than 0.15 indicated that addition of an ECG (n+1) would significantly change the NF and should be included in the analysis [12]. The NF of the stable ECGs in the OS epithelial regions (table 3) was used to normalise the linear Cp values of the genes of interest. The ECGs and the genes of interest were quantified with the same batch of cDNA. The Cp values of genes of interest were transformed to linear values similar to ECGs. These linear values were then normalised with NF (table 4), with the following formula:
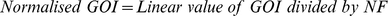



**Table 2:** Cp values of endogenous control genes in ocular surface epithelial regions.

	Cornea 1	Cornea 2	Cornea 3	Mean ± SEM	STDEV
B2M	**24.08**	**24.03**	**24.11**	**24.07±0.02**	**0.04**
GUSB	**21.52**	**21.48**	**21.57**	**21.52±0.03**	**0.04**
GAPDH	**25.97**	**25.97**	**25.86**	**25.93±0.04**	**0.06**
HPRT1	**26.32**	**26.21**	**26.17**	**26.23±0.04**	**0.08**
TBP	**21.55**	**21.46**	**21.36**	**21.46±0.05**	**0.09**
PGK1	**27.04**	**26.81**	**26.83**	**26.89±0.07**	**0.13**
RPLP0	**26.74**	**26.59**	**25.85**	**26.39±0.27**	**0.48**
18s	**13.07**	**14.09**	**14.03**	**13.73±0.33**	**0.57**
ACTB	**28.46**	**28.29**	**27.04**	**27.93±0.45**	**0.77**
PPIA	**15.08**	**16.9**	**15.07**	**15.68±0.61**	**1.05**

	Limbus 1	Limbus 2	Limbus3	Mean ± SEM	STDEV
TBP	**25.85**	**25.88**	**25.87**	**25.87±0.01**	**0.01**
GUSB	**22.59**	**22.56**	**22.57**	**22.57±0.01**	**0.01**
PPIA	**15.13**	**15.11**	**15.15**	**15.13±0.01**	**0.02**
HPRT1	**24.44**	**24.53**	**24.49**	**24.49±0.03**	**0.04**
18s	**12.29**	**12.37**	**12.24**	**12.3±0.04**	**0.06**
PGK1	**24.72**	**24.49**	**24.92**	**24.71±0.12**	**0.21**
GAPDH	**26.58**	**26.09**	**26.64**	**26.44±0.17**	**0.3**
RPLP0	**23.16**	**23.89**	**23.26**	**23.44±0.23**	**0.39**
ACTB	**31.84**	**33.57**	**31.9**	**32.44±0.57**	**0.98**
B2M	**18.12**	**19.97**	**18.31**	**18.8±0.59**	**1.02**

	LEC 1	LEC 2	LEC 3	Mean ± SEM	STDEV
ACTB	**29.74**	**29.83**	**29.9**	**29.82±0.05**	**0.08**
HPRT1	**29.35**	**29.44**	**29.28**	**29.36±0.05**	**0.08**
TBP	**30.71**	**30.68**	**30.87**	**30.75±0.06**	**0.1**
GAPDH	**29.11**	**28.94**	**29.21**	**29.09±0.08**	**0.14**
PPIA	**15.43**	**15.73**	**15.72**	**15.67±0.09**	**0.17**
B2M	**20.76**	**21.12**	**20.97**	**20.95±0.10**	**0.18**
GUSB	**27.57**	**27.14**	**27.01**	**27.24±0.17**	**0.29**
18s	**15.83**	**16.63**	**15.77**	**16.08±0.28**	**0.48**
RPLP0	**26.19**	**26.88**	**25.87**	**26.31±0.30**	**0.52**
PGK1	**31.07**	**32.18**	**32.59**	**31.97±0.45**	**0.79**

	Conj 1	Conj 2	Conj 3	Mean ± SEM	STDEV
RPLP0	**23.06**	**22.94**	**22.98**	**22.99±0.03**	**0.06**
TBP	**25.91**	**25.72**	**25.76**	**25.8±0.06**	**0.1**
B2M	**16.88**	**16.74**	**16.95**	**16.86±0.06**	**0.11**
ACTB	**33.61**	**34.04**	**33.63**	**33.76±0.14**	**0.24**
GAPDH	**22**	**22.71**	**22.4**	**22.37±0.20**	**0.35**
HPRT1	**19.04**	**18.75**	**19.47**	**19.09±0.21**	**0.36**
PPIA	**12.83**	**12.89**	**12.22**	**12.65±0.21**	**0.37**
PGK1	**20.12**	**19.95**	**20.88**	**20.32±0.29**	**0.49**
18s	**17.62**	**17.69**	**16.05**	**17.12±0.53**	**0.93**
GUSB	**19.07**	**19.51**	**20.92**	**19.83±0.56**	**0.96**

Abbreviations: LEC: limbal epithelial crypt, Conj: conjunctiva.

**Table 3:** Normalisation factor calculated with stable pair of endogenous control genes.

	PPIA	RPLPO	Normalisation Factor
**Cornea 1**	0.137738	0.071794	0.456256388
**Cornea 2**	0.03901	0.07966	0.255769594
**Cornea 3**	0.138376	0.133046	0.622544988
**Limbus 1**	0.133046	0.858565	1.550696839
**Limbus 2**	0.135216	0.517632	1.213844261
**Limbus 3**	0.130912	0.80107	1.485810368
**LEC 1**	0.108317	0.105112	0.489568736
**LEC 2**	0.087778	0.065154	0.346978137
**LEC 3**	0.088184	0.131215	0.493543789
**Conj 1**	0.656712	0.920188	3.566683831
**Conj 2**	0.628507	1	3.637422894
**Conj 3**	1	0.972655	4.524996468
**M<1.17**	**1.166197**	**1.166197**	

Abbreviations: LEC: limbal epithelial crypt, Conj: conjunctiva.

**Table 4:** Ranking of the endogenous control genes with increasing stability in descending order in ocular surface regions.

Ocular surface regions				
All regions	Cornea	Limbus	LEC	Conjunctiva
*ACTB*	*PPIA*	*B2M*	*PGK1*	*18s*
*18s*	*ACTB*	*ACTB*	*RPLP0*	*GUSB*
*TBP*	*18s*	*RPLP0*	*18s*	*PGK1*
*B2M*	*RPLP0*	*GAPDH*	*GUSB*	*HPRT*
*PGK1*	*PGK1*	*PGK1*	*GAPDH*	*PPIA*
*HPRT*	*GUSB*	*18s*	*HPRT*	*GAPDH*
*GUSB*	*B2M*	*HPRT*	*TBP*	*ACTB*
*GAPDH*	*GAPDH*	*TBP*	*ACTB*	*B2M*
*PPIA- RPLP0*	*HPRT- TBP*	*GUSB- PPIA*	*B2M -PPIA*	*RPLP0- TBP*
**M = 1.16–3.20**	***M = 0.02–0.53***	***M = 0.02–0.50***	***M = 0.07–0.42***	***M = 0.03–0.63***

M value represents the average expression stability of the endogenous control genes pairs. Abbreviations used in the table: LEC: limbal epithelial crypt, Conj: conjunctiva.

## Results

**Figure 1:**
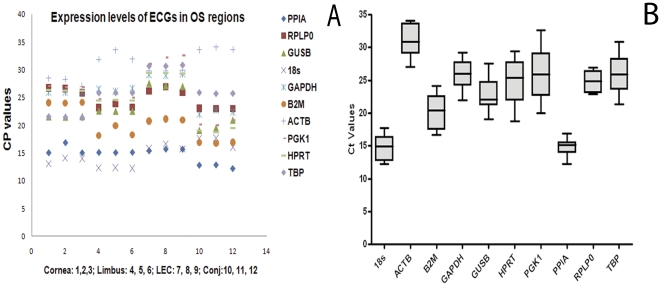
Distribution of cp values of ECGs in different OS epithelial regions. Figure 1A, the scatter graph of the Cp values of all the ECGs shows all the ECGs had cp value≤35. The order of samples 1–12 were as follows: Samples 1, 2, 3 were cornea 1, cornea 2, cornea 3, samples 4, 5, 6 were limbus 1, limbus 2, limbus 3, samples 7, 8, 9 were LEC 1, LEC 2, LEC 3 and samples 10, 11, 12 were conjunctiva 1, conjunctiva 2, conjunctiva 3. Figure 1B is a box plot of q PCR cp values of the ECGs. Each box plot is mean Cp value of all OS epithelial regions. The median Cp value is represented as black line within the box plot, and it divides the Cp values into lower and upper quartile ranges. The whiskers represent the upper and lower data range for the samples tested. The box plot of PPIA demonstrates tight distribution of the samples around the median value. Similarly box plot of RPLP0 shows uniform distribution of Cp values around the median with tight whisker distribution.

The expression level of the 10 ECG were determined in triplicates of 12 samples of OS epithelial regions obtained from three pairs of human cadaver eyes. Samples with a standard deviation <0.3 from the mean Cp of the triplicates were considered for further analysis. The Cp values of ten ECGs were plotted to compare transcription levels of these genes in OS epithelial regions of cornea, limbus, LEC and conjunctiva (figure 1 A, B). The raw expression values or the Cp values of all ECGs at the OS epithelial regions ranged from 12 to 33, which was below the cut-off value of 35 (table 2, figure 1 A, B). TBP, PGK1, GAPDH had most variable expression in LEC. Limbus and conjunctiva had higher Cp values for ACTB compared to other regions. PPIA and RPLP0 were found to have the most uniform expression pattern across the entire OS epithelial regions (figure 1). Box plot graph for the endogenous genes expression in OS epithelial samples showed tight distribution of the samples for PPIA and RPLP0 around the median Cp values of 15 and 25 respectively (figure 1B).

### Expression values of ECGs

The ECGs exhibited wide range of expression and were grouped into high, moderate and low expression categories based on mean Cp values. We observed that the PPIA was abundantly expressed in LEC, limbus, cornea and conjunctiva with narrowest range of Cp values (12.65±0.21 to 15.68±0.61). As seen in figure 1, LEC had the least level of expression of most of the ECGs such as TBP, HPRT1, GAPDH, ACTB, and PGK1 (Cp values: 27 to 33) compared to other regions. Whilst the conjunctiva had shown the most abundant expression of HPRT1, PPIA, GUSB, 18S, B2M with Cp values ranging from 12 to 19. In cornea and limbus the majority of the ECGs (GUSB, HPRT1, TBP, GAPDH, PGK1, and RPLP0) were moderately expressed with Cp values ranging from 20 to 26.

### Average expression stability and ranking of the ECGs

**Figure 2:**
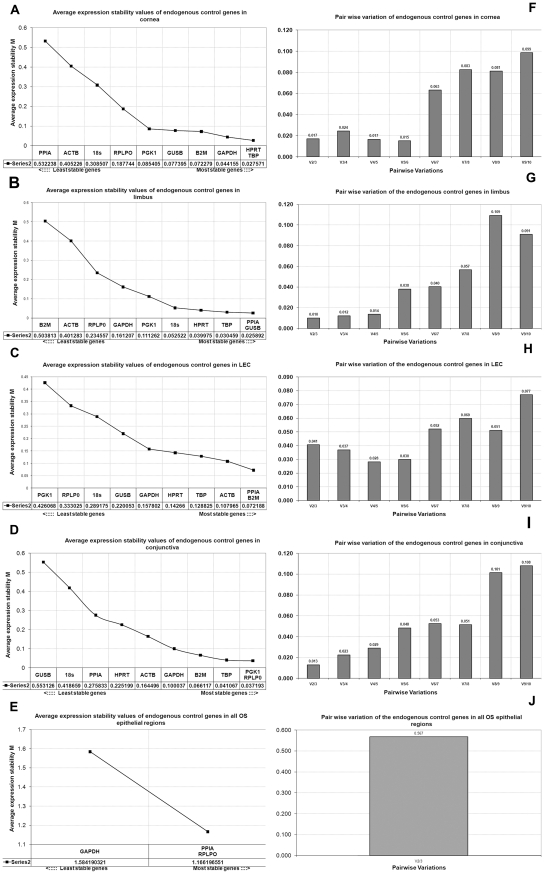
Average expression stability values of endogenous control genes in ocular surface regions. The M values on the Y axis measures gene expression stability. Higher the M value the lower is the gene stability and vice versa. In graphs A, B, C, D, E the gene stability is represented in increasing order of stability from left to right. In graphs F, G, H, I, J, pair wise variation is represented. The pair wise variation for V_2/3_ is less than 0.15 for all the regions represented by graphs F, G, H, I and J. Hence Normalisation Factor (NF) calculated with these two genes is stable and would not significantly change with addition of a third endogenous control gene.

GeNorm ranked the ECGs based on their expression stability and identified several stable genes, of which, one pair was the most stable and could be considered for normalisation of qPCR data for genes of interest for the OS. The stability ranking of the genes across all OS epithelial regions from least to most stable were: ACTB<18S<TBP<B2M<PGK1<HPRT<GUSB<GAPDH<PPIA and RPLP0 with M value 1.16 (below the threshold value of 1.5). For individual OS epithelial regions, the most stable ECGs pairs were: cornea HPRT1-TBP, limbus GUSB-PPIA, LEC B2M-PPIA, conjunctiva RPLP0-TBP (table 4, figure 2). Pair-wise variation was performed to determine optimal number of ECGs required for normalisation. The pairwise variation in cornea, limbus, LEC and conjunctiva respectively was shown in Figure 2F–2I. Graph bar ‘V_2/3_’ in these regions had values less than 0.15, therefore, addition of other ECGs to the stable pair of ECG was not necessary for calculation of NF. Although ‘V_2/3_’ for ECGs in all OS epithelial regions was 0.567, previous sequentially ranked ECG (GAPDH) was not included in the calculation of NF because it was an unstable gene with M value (1.584), slightly higher than the cut off of 1.5 (figure 2 E, 2J). Therefore, only the most stable pair of ECGs for all the OS epithelial regions (PPIA-RPLP0) (figure 2E, 2J) was considered in the calculation of the NF (table 3).

### Normalisation of genes of interest (GOI)

**Figure 3:**
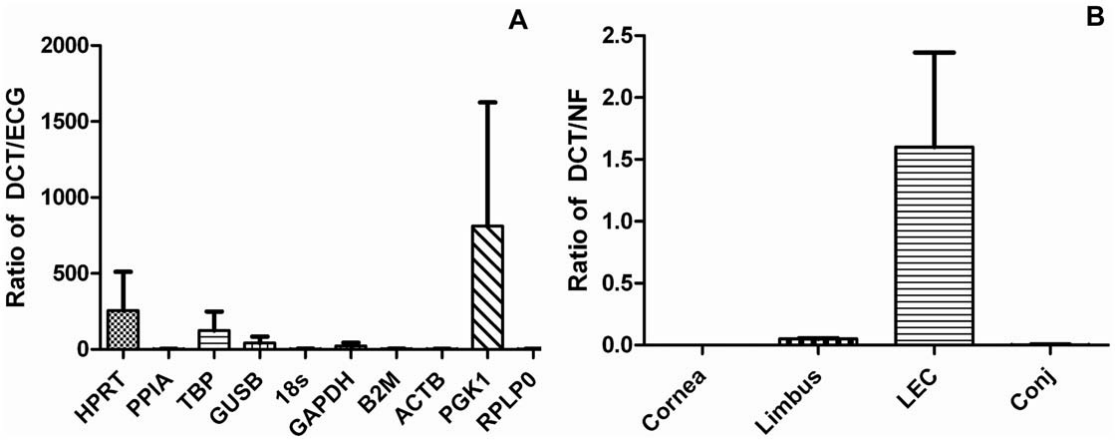
Normalised gene expression of Dopachrome Tautomerase (DCT) in ocular surface regions. Graph A shows the normalised expression of Dopachrome Tautomerase (DCT) in OS epithelial regions using the 10 individual ECGs studied. As seen in graph A, DCT was found to have highly variable expression with individual endogenous control genes. Graph B shows normalised expression of DCT using the normalisation factor (NF) as determined in this study. Normalised expression of DCT with NF is stable and some expression of DCT is also noted in limbus.

The gene of interest in OS epithelial regions (DCT) was normalised individually with the ECGs (figure 3A) and with NF (figure 3B), to demonstrate the effect of ECGs on the gene of interest. The results show DCT expressed in LEC unlike other regions. As compared to normalised expression of DCT with NF, variation in normalised expression of DCT was seen with individual ECGs (figure 3A). Marked variation in normalised expression of DCT with individual ECGs was noted (figure 3A) as compared to normalization with NF in which the DCT expression showed higher fold change expression (figure 3B).

## Discussion

The ocular stem cell research has in recent past become the key focus of various vision research groups and it is believed that OS stem cells reside in the limbal epithelial crypt (LEC) [2], [23], [26]–[28]. The LEC derived stem cells were responsible for replenishing the corneal surface following ocular surface disease or injury such as chemical burns [29]. Molecular biology techniques, in particular both microarray and quantitative gene expression (qPCR) analyses of the corneal epithelium, are being widely used to ascertain subpopulations of cells with different functionality. It is therefore important that the endogenous controls are standardised for consistency in published reports. Previous studies have shown that selection of appropriate ECGs is crucial for accuracy of gene normalisation strategy in qPCR [30]. Use of inappropriate ECGs would lead to inappropriate normalisation of the qPCR data because the expression of these reference control genes may be variably expressed between different tissues and experimental conditions [31], [32]. With LMD, extraction of pure samples of cell populations and tissues can be achieved from heterogeneous tissues, leading to improved accuracy of gene expression profiling [33]. Similarly, we had collected pure samples of OS epithelial regions using LMD, avoiding contamination from the surrounding tissues. This technique was especially suitable for obtaining LEC tissue, which is deeply seated in the stromal tissue and could not be separated by mechanical dissection. However, RNA yield of the laser microdissected epithelial samples is minimal and poses a limitation for multiple gene profiling studies. A recent report on the amplification of laser microdissected RNA tissue with Ribo-SPIA amplification procedure (Nugen Technologies, Ovation kit, San Carlos, CA) has shown to result in sufficient yield of amplified cDNA with increased sensitivity in detection of low expressed genes [34]. We had similarly processed and amplified the laser microdissected RNA samples for qPCR experiments on a panel of ECGs and genes of interest in the OS epithelial tissues. In this process expression of 18S rRNA was also noted in the cDNA samples. This could be explained with the action of the NuGEN amplification system which uses a primer mix of oligo dTs and random primers hybridised either to 5′ portion of the poly A sequence or randomly across the transcript. 18S rRNA comprised 20% of the tRNA samples used. Hence 18S rRNA is present in proportionately larger quantity in the cell compared to mRNA. Therefore random primers in the NuGEN WT-Ovation™ Pico RNA Amplification System opportunistically prime 18S rRNA to generate cDNA. This explains the observed results of amplification of 18S rRNA. Ideally, the ECG should be stably expressed in various tissues and experimental conditions [8]. However, we had noted variations in the quantitative gene expression in a panel of ECGs such as ACTB, HPRT1, PGK1, TBP, and GAPDH across the OS epithelial regions. This variation was prominent in LEC compared to other regions, wherein; most of the ECGs had high Cp values. In our previous study, we have shown that LEC is a quiescent region in comparison to cornea, limbus and conjunctiva; therefore the expression of ECGs which are mainly involved in metabolic processes could be reduced in LEC [2]. We had identified stably expressed ECGs in OS epithelial regions with the use of geNorm algorithm, which was described and validated by Vandesompele and co-workers for efficient normalisation of qPCR data. Our results in current study have demonstrated PPIA-RPLP0 pair to be most stable across all regions with M value 1.16 (well below the cutoff 1.50) [12]. Therefore, this stable ECGs pair; PPIA-RPLP0 was used to calculate the normalisation factor for all the OS epithelial regions. The stable ECG pair for each OS epithelial regions was found to be HPRT1-TBP in cornea, GUSB-PPIA in limbus, B2M-PPIA and RPLP0-PGK1 in LEC and conjunctiva respectively. Therefore, for comparison of different OS regions, the PPIA-RPLP0 should be the preferred ECG pair; however, when each OS region is studied individually, the above mentioned ECG pairs could be selected accordingly. Vandosompele group have suggested the use of pair wise variation for determination of number of ECGs suitable for calculation of normalisation factor in GeNorm analysis [12]. In our study, pair wise variations showed insignificant effect of addition of third ECGs to the NF. We have demonstrated variable expression of panel of ECGs in OS epithelial regions by normalisation of gene of interest (DCT) in LEC with these ECGs individually. However normalisation with NF, which was more sensitive as a low level of expression, was also detected in limbus. Pubmed search of the published literature have shown GAPDH [35]–[37] and ACTB [38]–[40] as popularly used ECGs for qPCR experiments in ocular surface epithelium. However, in this study these genes were found to have variable expression across the OS epithelial regions and the normalised expression of DCT with GAPDH was minimal in LEC and absent with ACTB as compared to the NF.

We therefore, propose the use of PPIA-RPLP0 pair as reference genes in comparative qPCR involving different OS epithelial regions in future studies. Similarly, HPRT1-TBP, GUSB-PPIA, B2M-PPIA and RPLP0-TBP ECG pairs could be considered when individually studying cornea, LEC, limbus and conjunctiva OS epithelial regions respectively. To this end, this is the first study that identified the expression of stable ECGs in LMD OS epithelial regions namely LEC, cornea, limbus and conjunctiva. Efficient normalisation of the genes of interest was achieved with the NF derived from this stable pair of ECGs. This study has also identified that the combination of ECGs (PPIA-RPLP0) has more accurately normalised qPCR data in laser microdissected human OS epithelial tissues. For future studies on OS tissues obtained by means other than laser microdissection, such as cell cultures, these reported genes may serve as reference but it is anticipated that geometric mean of their Cp values may need to be calculated for the specific experiment as the species variation, sample types, experimental conditions may influence the expression stability values and the NF.
